# 2-(1*H*-Benzimidazol-1-yl)-1-(2-furyl)ethanone *O*-propyloxime

**DOI:** 10.1107/S1600536809020844

**Published:** 2009-06-06

**Authors:** Özden Özel Güven, Taner Erdoğan, Simon J. Coles, Tuncer Hökelek

**Affiliations:** a Department of Chemistry, Zonguldak Karaelmas University, 67100 Zonguldak, Turkey; b Department of Chemistry, Southampton University, SO17 1BJ Southampton, England; c Department of Physics, Hacettepe University, 06800 Beytepe, Ankara, Turkey

## Abstract

In the mol­ecule of the title compound, C_16_H_17_N_3_O_2_, the planar benzimidazole ring system  [maximum deviation = 0.013 (1) Å] is oriented at a dihedral angle of 75.32 (4)° with respect to the furan ring. An intra­molecular C—H⋯O inter­action results in the formation of a planar six-membered ring [maximum deviation = 0.019 (15) Å], which is oriented at a dihedral angle of 1.91 (3)° with respect to the adjacent furan ring. In the crystal structure, inter­molecular C—H⋯N inter­actions link the mol­ecules into centrosymmetric *R*_2_^2^(18) dimers. In addition, the structure is stabilized by π–π contacts between the imidazole rings [centroid–centroid distance = 3.5307 (8) Å] and weak C—H⋯π inter­actions.

## Related literature

Several compounds containing an oxime or an oxime ether function have been reported to exhibit anti­microbial activity, see: Baji *et al.* (1995 ▶); Bhandari *et al.* (2009 ▶); Emami *et al.* (2002 ▶, 2004 ▶); Milanese *et al.* (2007 ▶); Polak (1982 ▶); Poretta *et al.* (1993 ▶); Ramalingan *et al.* (2006 ▶); Rosello *et al.* (2002 ▶). For related structures, see: Özel Güven *et al.* (2007*a* ▶,*b* ▶). For ring motifs, see: Bernstein *et al.* (1995 ▶).
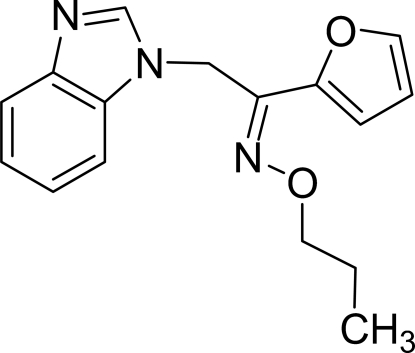


## Experimental

### 

#### Crystal data

C_16_H_17_N_3_O_2_
*M* *_r_* = 283.33
Monoclinic, 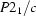

*a* = 8.4164 (2) Å
*b* = 18.2524 (3) Å
*c* = 10.2246 (2) Å
β = 111.354 (1)°
*V* = 1462.87 (5) Å^3^
*Z* = 4
Mo *K*α radiation
μ = 0.09 mm^−1^
*T* = 120 K
0.50 × 0.35 × 0.35 mm

#### Data collection

Bruker–Nonius Kappa CCD diffractometer
Absorption correction: multi-scan (*SADABS*; Sheldrick, 2007 ▶) *T*_min_ = 0.958, *T*_max_ = 0.960
19714 measured reflections
3344 independent reflections
2755 reflections with *I* > 2σ(*I*)
*R*_int_ = 0.038

#### Refinement

*R*[*F* ^2^ > 2σ(*F* ^2^)] = 0.043
*wR*(*F* ^2^) = 0.114
*S* = 1.14
3344 reflections
259 parameters
All H-atom parameters refined
Δρ_max_ = 0.37 e Å^−3^
Δρ_min_ = −0.36 e Å^−3^

### 

Data collection: *COLLECT* (Hooft, 1998 ▶); cell refinement: *DENZO* (Otwinowski & Minor, 1997 ▶) and *COLLECT*; data reduction: *DENZO* nd *COLLECT*; program(s) used to solve structure: *SHELXS97* (Sheldrick, 2008 ▶); program(s) used to refine structure: *SHELXL97* (Sheldrick, 2008 ▶); molecular graphics: *ORTEP-3 for Windows* (Farrugia, 1997 ▶); software used to prepare material for publication: *WinGX* (Farrugia, 1999 ▶) and *PLATON* (Spek, 2009 ▶).

## Supplementary Material

Crystal structure: contains datablocks I, global. DOI: 10.1107/S1600536809020844/ci2817sup1.cif

Structure factors: contains datablocks I. DOI: 10.1107/S1600536809020844/ci2817Isup2.hkl

Additional supplementary materials:  crystallographic information; 3D view; checkCIF report

## Figures and Tables

**Table 1:** Hydrogen-bond geometry (Å, °)

*D*—H⋯*A*	*D*—H	H⋯*A*	*D*⋯*A*	*D*—H⋯*A*
C11—H11⋯O2	0.97 (2)	2.359 (16)	2.7930 (17)	106 (1)
C13—H13⋯N2^i^	0.96 (2)	2.360 (15)	3.2872 (17)	162 (1)
C14—H141⋯*Cg*1^ii^	0.99 (2)	2.968 (17)	3.8346 (16)	146 (1)
C16—H161⋯*Cg*2^ii^	0.99 (2)	2.685 (18)	3.5644 (16)	148 (1)
C16—H163⋯*Cg*3^ii^	1.00 (2)	2.643 (18)	3.5901 (15)	157 (1)

Symmetry codes: (i) 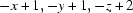; (ii) 
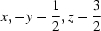
. *Cg*1, *Cg*2 and *Cg*3 are the centroids of the C2–C7, N1/N2/C1/C2/C7 and O1/C10–C13 rings, respectively.

**Fig. 1.**
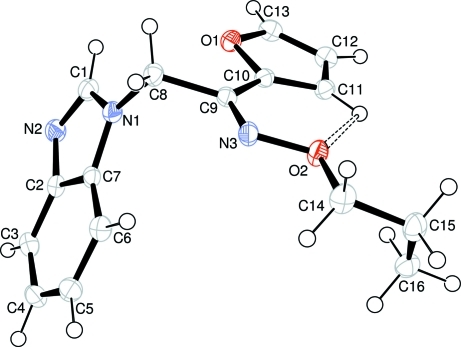
The molecular structure of the title compound, with the atom-numbering scheme. Displacement ellipsoids are drawn at the 50% probability level. The dashed line indicates a hydrogen bond.

**Fig. 2.**
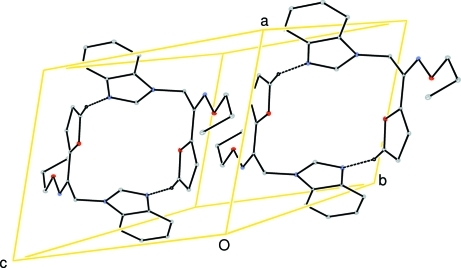
A partial packing diagram of the title compound. Hydrogen bonds are shown as dashed lines. H atoms not involved in hydrogen bonding have been omitted for clarity.

## supplementary crystallographic information

### Comment

Oxiconazole (Polak, 1982) is a well known antifungal agent with a broad spectrum of activity, used for the treatment of skin mycoses. It is structurally characterized by an oxime ether group. Several compounds containing an oxime or an oxime ether function have been reported to exhibit antimicrobial activity (Poretta *et al.*, 1993; Baji *et al.*, 1995; Rosello *et al.*, 2002; Emami *et al.*, 2002; Emami *et al.*, 2004; Ramalingan *et al.*, 2006; Milanese *et al.*, 2007; Bhandari *et al.*, 2009). In our earlier studies, we reported the crystal structure of a benzimidazole substituted oxiconazole derivative (Özel Güven *et al.*, 2007*a*). Now, we report herein the crystal structure of the title alkyl oxime ether.

In the molecule of the title compound (Fig. 1), the bond lengths and angles are generally within normal ranges. The planar benzimidazole ring system is oriented with respect to the furan ring at a dihedral angle of 75.32 (4)°. Atoms O2, N3, C8, C9, C14 and C15 are 0.073 (1), 0.024 (1), -0.064 (1), -0.009 (1), 0.088 (1) and -0.082 (1) Å away from the furan ring plane, respectively, while atom C8 is at a distance of -0.007 (1) Å to the benzimidazole ring plane. So, they are coplanar with the adjacent rings. The N1—C1—N2 [114.29 (11)°], N2—C2—C7 [110.24 (11)°] and C2—C7—C6 [122.57 (11)°] bond angles are enlarged, while C5—C6—C7 [116.36 (11)°] and C2—C3—C4 [117.87 (11)°] bond angles are narrowed. An intramolecular C—H···O interaction (Table 1) results in the formation of a planar six-membered ring, (O2/N3/C9—C11/H11), which is oriented with respect to the adjacent furan ring at a dihedral angle of 1.91 (3)°. So, they are almost coplanar.

In the crystal structure, intermolecular C—H···N interactions (Table 1) link the molecules into centrosymmetric dimers through *R*_2_^2^(18) ring motifs (Bernstein *et al.*, 1995) (Fig. 2), in which they may be effective in the stabilization of the structure. The π···π contact between the benzimidazole rings [*Cg*2···*Cg*2^i^ = 3.5307 (8) Å, where *Cg*1 is centroid of the N1/N2/C1/C2/C7 ring; symmetry code: (i) 2 - *x*, 1 -*y*, 2 - *z*] may further stabilize the structure. There also exist three weak C—H···π interactions (Table 1).

### Experimental

The title compound was synthesized by the reaction of 2-(1*H*-benzimidazol-1-yl)-1-(furan-2-yl)ethanone oxime obtained from 2-(1*H*-benzimidazol-1-yl)-1-(furan-2-yl)ethanone (Özel Güven *et al.*, 2007*b*) with n-propyl bromide and NaH. To a solution of 2-(1*H*-benzimidazol-1-yl)-1-(furan-2-yl)ethanone oxime (350 mg, 1.451 mmol) in DMF (5 ml) was added NaH (58 mg, 1.451 mmol) in small fractions. Then, n-propyl bromide (178 mg, 1.451 mmol) was added dropwise. The mixture was stirred at room temperature for 3 h and the excess of hydride was decomposed with a small amount of methyl alcohol. After evaporation to dryness under reduced pressure, the crude residue was suspended with water and extracted with methylene chloride. The organic layer was dried over anhydrous sodium sulfate and then evaporated to dryness. The crude product was purified by chromatography on a silica-gel column using chloroform and recrystallized from hexane-ethyl acetate (1:3) mixture to obtain pale-yellowish crystals (yield 150 mg, 37%).

### Refinement

H atoms were located in a difference map and were refined isotropically [C—H = 0.959 (15)–1.015 (17) Å and *U*_iso_(H) = 0.018 (3)–0.048 (5) Å^2^].

### Crystal data

**Table:**

C_16_H_17_N_3_O_2_	*F*(000) = 600
*M**_r_* = 283.33	*D*_x_ = 1.286 Mg m^−^^3^
Monoclinic, *P*2_1_/*c*	Mo *K*α radiation, λ = 0.71073 Å
Hall symbol: -P 2ybc	Cell parameters from 3395 reflections
*a* = 8.4164 (2) Å	θ = 2.9–27.5°
*b* = 18.2524 (3) Å	µ = 0.09 mm^−^^1^
*c* = 10.2246 (2) Å	*T* = 120 K
β = 111.354 (1)°	Block, pale yellow
*V* = 1462.87 (5) Å^3^	0.50 × 0.35 × 0.35 mm
*Z* = 4	

### Data collection

**Table:**

Bruker–Nonius Kappa CCD diffractometer	3344 independent reflections
Radiation source: fine-focus sealed tube	2755 reflections with *I* > 2σ(*I*)
graphite	*R*_int_ = 0.038
Detector resolution: 9.091 pixels mm^-1^	θ_max_ = 27.5°, θ_min_ = 2.9°
φ & ω scans	*h* = −10→10
Absorption correction: multi-scan (*SADABS*; Sheldrick, 2007)	*k* = −23→23
*T*_min_ = 0.958, *T*_max_ = 0.960	*l* = −13→13
19714 measured reflections	

### Refinement

**Table:**

Refinement on *F*^2^	Secondary atom site location: difference Fourier map
Least-squares matrix: full	Hydrogen site location: inferred from neighbouring sites
*R*[*F*^2^ > 2σ(*F*^2^)] = 0.043	All H-atom parameters refined
*wR*(*F*^2^) = 0.114	*w* = 1/[σ^2^(*F*_o_^2^) + (0.0594*P*)^2^ + 0.2758*P*] where *P* = (*F*_o_^2^ + 2*F*_c_^2^)/3
*S* = 1.14	(Δ/σ)_max_ = 0.001
3344 reflections	Δρ_max_ = 0.37 e Å^−^^3^
259 parameters	Δρ_min_ = −0.36 e Å^−^^3^
0 restraints	Extinction correction: *SHELXL97*, Fc^*^=kFc[1+0.001xFc^2^λ^3^/sin(2θ)]^-1/4^
Primary atom site location: structure-invariant direct methods	Extinction coefficient: 0.065 (5)

### Special details

**Table:**

Geometry. All e.s.d.'s (except the e.s.d. in the dihedral angle between two l.s. planes) are estimated using the full covariance matrix. The cell e.s.d.'s are taken into account individually in the estimation of e.s.d.'s in distances, angles and torsion angles; correlations between e.s.d.'s in cell parameters are only used when they are defined by crystal symmetry. An approximate (isotropic) treatment of cell e.s.d.'s is used for estimating e.s.d.'s involving l.s. planes.
Refinement. Refinement of *F*^2^ against ALL reflections. The weighted *R*-factor *wR* and goodness of fit *S* are based on *F*^2^, conventional *R*-factors *R* are based on *F*, with *F* set to zero for negative *F*^2^. The threshold expression of *F*^2^ > σ(*F*^2^) is used only for calculating *R*-factors(gt) *etc*. and is not relevant to the choice of reflections for refinement. *R*-factors based on *F*^2^ are statistically about twice as large as those based on *F*, and *R*- factors based on ALL data will be even larger.

### Fractional atomic coordinates and isotropic or equivalent isotropic displacement parameters (Å^2^)

**Table:**

	*x*	*y*	*z*	*U*_iso_*/*U*_eq_	
O1	0.43354 (11)	0.47469 (5)	0.69234 (9)	0.0210 (2)	
O2	0.62124 (10)	0.35487 (5)	0.44862 (9)	0.0215 (2)	
N1	0.81649 (13)	0.45397 (6)	0.87655 (10)	0.0191 (2)	
N2	0.81640 (14)	0.44200 (6)	1.09515 (11)	0.0234 (3)	
N3	0.73483 (13)	0.39962 (6)	0.55091 (11)	0.0203 (2)	
C1	0.75737 (16)	0.47823 (7)	0.97642 (13)	0.0226 (3)	
H1	0.680 (2)	0.5192 (8)	0.9590 (16)	0.025 (4)*	
C2	0.92279 (15)	0.38916 (7)	1.07227 (12)	0.0196 (3)	
C3	1.01939 (16)	0.33471 (7)	1.16205 (13)	0.0229 (3)	
H3	1.016 (2)	0.3284 (9)	1.2569 (17)	0.034 (4)*	
C4	1.11688 (16)	0.28954 (7)	1.11299 (14)	0.0250 (3)	
H4	1.185 (2)	0.2500 (9)	1.1699 (17)	0.035 (4)*	
C5	1.11963 (16)	0.29780 (7)	0.97705 (14)	0.0249 (3)	
H5	1.192 (2)	0.2655 (9)	0.9461 (16)	0.033 (4)*	
C6	1.02292 (16)	0.35075 (7)	0.88540 (13)	0.0223 (3)	
H6	1.0243 (19)	0.3573 (8)	0.7909 (16)	0.026 (4)*	
C7	0.92492 (15)	0.39601 (6)	0.93600 (12)	0.0180 (3)	
C8	0.77611 (17)	0.48381 (7)	0.73516 (13)	0.0209 (3)	
H81	0.8843 (18)	0.4894 (8)	0.7176 (14)	0.018 (3)*	
H82	0.7235 (19)	0.5324 (8)	0.7319 (15)	0.021 (4)*	
C9	0.65977 (15)	0.43432 (6)	0.62241 (12)	0.0177 (3)	
C10	0.48021 (15)	0.42984 (6)	0.60389 (12)	0.0176 (3)	
C11	0.34138 (16)	0.39130 (7)	0.52080 (13)	0.0195 (3)	
H11	0.3406 (18)	0.3571 (8)	0.4478 (15)	0.024 (4)*	
C12	0.20149 (17)	0.41338 (7)	0.55880 (14)	0.0228 (3)	
H12	0.081 (2)	0.3957 (8)	0.5195 (16)	0.027 (4)*	
C13	0.26348 (17)	0.46348 (7)	0.66207 (13)	0.0233 (3)	
H13	0.2152 (18)	0.4915 (8)	0.7179 (15)	0.021 (3)*	
C14	0.71245 (17)	0.31673 (8)	0.37379 (15)	0.0269 (3)	
H141	0.813 (2)	0.2925 (9)	0.4441 (17)	0.032 (4)*	
H142	0.749 (2)	0.3531 (9)	0.3176 (17)	0.034 (4)*	
C15	0.59066 (18)	0.26057 (8)	0.28123 (14)	0.0278 (3)	
H151	0.650 (2)	0.2349 (10)	0.2257 (17)	0.040 (4)*	
H152	0.493 (2)	0.2866 (9)	0.2113 (18)	0.040 (5)*	
C16	0.52829 (18)	0.20602 (8)	0.36374 (15)	0.0287 (3)	
H161	0.624 (2)	0.1794 (10)	0.4348 (19)	0.048 (5)*	
H162	0.468 (2)	0.2317 (9)	0.4205 (16)	0.036 (4)*	
H163	0.449 (2)	0.1687 (10)	0.3015 (19)	0.047 (5)*	

### Atomic displacement parameters (Å^2^)

**Table:**

	*U*^11^	*U*^22^	*U*^33^	*U*^12^	*U*^13^	*U*^23^
O1	0.0239 (5)	0.0207 (4)	0.0187 (4)	0.0014 (3)	0.0082 (4)	−0.0027 (3)
O2	0.0178 (4)	0.0252 (5)	0.0212 (4)	−0.0019 (3)	0.0069 (4)	−0.0078 (4)
N1	0.0206 (5)	0.0191 (5)	0.0163 (5)	−0.0009 (4)	0.0052 (4)	−0.0009 (4)
N2	0.0248 (6)	0.0258 (6)	0.0187 (5)	0.0015 (4)	0.0066 (4)	−0.0034 (4)
N3	0.0201 (5)	0.0212 (5)	0.0169 (5)	−0.0026 (4)	0.0036 (4)	−0.0013 (4)
C1	0.0234 (6)	0.0223 (6)	0.0213 (6)	0.0001 (5)	0.0070 (5)	−0.0053 (5)
C2	0.0179 (6)	0.0221 (6)	0.0173 (6)	−0.0037 (5)	0.0047 (5)	−0.0036 (5)
C3	0.0202 (6)	0.0275 (7)	0.0185 (6)	−0.0021 (5)	0.0041 (5)	0.0012 (5)
C4	0.0191 (6)	0.0252 (7)	0.0261 (7)	0.0017 (5)	0.0026 (5)	0.0025 (5)
C5	0.0187 (6)	0.0264 (7)	0.0293 (7)	0.0016 (5)	0.0083 (5)	−0.0036 (5)
C6	0.0200 (6)	0.0266 (6)	0.0212 (6)	−0.0027 (5)	0.0086 (5)	−0.0022 (5)
C7	0.0155 (5)	0.0188 (6)	0.0178 (6)	−0.0035 (4)	0.0036 (5)	−0.0016 (4)
C8	0.0238 (6)	0.0200 (6)	0.0165 (6)	−0.0042 (5)	0.0045 (5)	0.0009 (5)
C9	0.0206 (6)	0.0161 (6)	0.0150 (6)	−0.0009 (4)	0.0050 (5)	0.0029 (4)
C10	0.0229 (6)	0.0157 (5)	0.0146 (5)	0.0013 (4)	0.0074 (5)	0.0010 (4)
C11	0.0219 (6)	0.0185 (6)	0.0181 (6)	−0.0005 (5)	0.0073 (5)	−0.0001 (5)
C12	0.0216 (6)	0.0240 (6)	0.0237 (6)	0.0010 (5)	0.0093 (5)	0.0018 (5)
C13	0.0235 (6)	0.0257 (6)	0.0235 (6)	0.0045 (5)	0.0116 (5)	0.0020 (5)
C14	0.0212 (7)	0.0350 (7)	0.0277 (7)	0.0011 (6)	0.0128 (6)	−0.0079 (6)
C15	0.0249 (7)	0.0354 (8)	0.0232 (6)	0.0032 (6)	0.0087 (6)	−0.0095 (6)
C16	0.0258 (7)	0.0267 (7)	0.0305 (7)	0.0041 (6)	0.0067 (6)	−0.0070 (6)

### Geometric parameters (Å, °)

**Table:**

O1—C10	1.3787 (14)	C6—H6	0.978 (15)
O1—C13	1.3652 (16)	C8—H81	0.996 (14)
O2—N3	1.3952 (13)	C8—H82	0.987 (15)
O2—C14	1.4447 (15)	C9—C8	1.5105 (16)
N1—C1	1.3625 (16)	C9—C10	1.4553 (16)
N1—C7	1.3847 (15)	C11—C10	1.3635 (17)
N1—C8	1.4643 (15)	C11—C12	1.4257 (17)
N2—C1	1.3110 (17)	C11—H11	0.971 (15)
N2—C2	1.3927 (16)	C12—H12	0.999 (16)
N3—C9	1.2927 (16)	C13—C12	1.3499 (19)
C1—H1	0.963 (15)	C13—H13	0.959 (15)
C2—C3	1.3956 (18)	C14—H141	0.994 (16)
C2—C7	1.4055 (17)	C14—H142	0.999 (17)
C3—H3	0.987 (16)	C15—C14	1.5136 (19)
C4—C3	1.3800 (18)	C15—C16	1.517 (2)
C4—C5	1.4066 (19)	C15—H151	0.999 (17)
C4—H4	0.973 (17)	C15—H152	0.994 (18)
C5—H5	0.982 (16)	C16—H161	0.993 (19)
C6—C5	1.3854 (18)	C16—H162	1.015 (17)
C6—C7	1.3933 (17)	C16—H163	1.001 (19)
			
C13—O1—C10	106.73 (9)	N3—C9—C10	126.66 (11)
N3—O2—C14	109.14 (9)	N3—C9—C8	114.21 (11)
C1—N1—C7	106.35 (10)	C10—C9—C8	119.13 (10)
C1—N1—C8	127.02 (11)	C11—C10—O1	109.32 (10)
C7—N1—C8	126.62 (10)	C11—C10—C9	136.37 (11)
C1—N2—C2	104.12 (10)	O1—C10—C9	114.31 (10)
C9—N3—O2	111.61 (10)	C10—C11—C12	106.84 (11)
N1—C1—H1	121.3 (9)	C10—C11—H11	125.1 (9)
N2—C1—N1	114.29 (11)	C12—C11—H11	128.0 (9)
N2—C1—H1	124.4 (9)	C13—C12—C11	106.40 (11)
N2—C2—C3	129.71 (11)	C13—C12—H12	125.5 (9)
N2—C2—C7	110.24 (11)	C11—C12—H12	128.1 (9)
C3—C2—C7	120.05 (11)	C12—C13—O1	110.70 (11)
C4—C3—C2	117.87 (11)	C12—C13—H13	134.4 (9)
C4—C3—H3	121.4 (10)	O1—C13—H13	114.9 (9)
C2—C3—H3	120.7 (10)	O2—C14—C15	106.74 (10)
C3—C4—C5	121.40 (12)	O2—C14—H142	108.7 (9)
C3—C4—H4	121.7 (9)	C15—C14—H142	111.9 (9)
C5—C4—H4	116.9 (9)	O2—C14—H141	108.0 (9)
C6—C5—C4	121.75 (12)	C15—C14—H141	110.9 (9)
C6—C5—H5	118.9 (9)	H142—C14—H141	110.5 (13)
C4—C5—H5	119.4 (9)	C14—C15—C16	112.93 (11)
C5—C6—C7	116.36 (11)	C14—C15—H151	107.9 (10)
C5—C6—H6	122.6 (9)	C16—C15—H151	110.7 (10)
C7—C6—H6	121.1 (9)	C14—C15—H152	108.7 (10)
N1—C7—C6	132.42 (11)	C16—C15—H152	110.5 (10)
N1—C7—C2	105.01 (10)	H151—C15—H152	105.7 (13)
C6—C7—C2	122.57 (11)	C15—C16—H161	112.0 (10)
N1—C8—C9	112.58 (9)	C15—C16—H162	111.2 (9)
N1—C8—H81	108.5 (8)	H161—C16—H162	104.4 (13)
C9—C8—H81	107.8 (8)	C15—C16—H163	112.1 (10)
N1—C8—H82	108.0 (8)	H161—C16—H163	107.8 (15)
C9—C8—H82	110.7 (9)	H162—C16—H163	108.8 (14)
H81—C8—H82	109.2 (12)		
			
C13—O1—C10—C11	−0.29 (13)	N2—C2—C7—N1	−0.36 (13)
C13—O1—C10—C9	−179.60 (10)	C3—C2—C7—N1	179.63 (11)
C10—O1—C13—C12	0.20 (13)	N2—C2—C7—C6	179.04 (11)
C14—O2—N3—C9	−179.10 (10)	C3—C2—C7—C6	−0.97 (18)
N3—O2—C14—C15	171.16 (10)	C5—C4—C3—C2	0.06 (19)
C7—N1—C1—N2	0.07 (15)	C3—C4—C5—C6	−1.0 (2)
C8—N1—C1—N2	−179.00 (11)	C7—C6—C5—C4	0.93 (18)
C1—N1—C7—C6	−179.14 (13)	C5—C6—C7—N1	179.26 (12)
C8—N1—C7—C6	−0.1 (2)	C5—C6—C7—C2	0.05 (18)
C1—N1—C7—C2	0.18 (13)	N3—C9—C8—N1	−106.07 (12)
C8—N1—C7—C2	179.25 (11)	C10—C9—C8—N1	73.98 (14)
C1—N1—C8—C9	−107.61 (14)	N3—C9—C10—C11	2.8 (2)
C7—N1—C8—C9	73.51 (15)	C8—C9—C10—C11	−177.25 (13)
C2—N2—C1—N1	−0.28 (14)	N3—C9—C10—O1	−178.13 (11)
C1—N2—C2—C3	−179.59 (13)	C8—C9—C10—O1	1.81 (15)
C1—N2—C2—C7	0.39 (13)	C12—C11—C10—O1	0.26 (13)
O2—N3—C9—C10	−0.07 (16)	C12—C11—C10—C9	179.35 (13)
O2—N3—C9—C8	179.98 (9)	C10—C11—C12—C13	−0.13 (14)
N2—C2—C3—C4	−179.13 (12)	O1—C13—C12—C11	−0.04 (14)
C7—C2—C3—C4	0.89 (18)	C16—C15—C14—O2	−59.43 (15)

### Hydrogen-bond geometry (Å, °)

**Table:**

*D*—H···*A*	*D*—H	H···*A*	*D*···*A*	*D*—H···*A*
C11—H11···O2	0.97 (2)	2.359 (16)	2.7930 (17)	106 (1)
C13—H13···N2^i^	0.96 (2)	2.360 (15)	3.2872 (17)	162 (1)
C14—H141···Cg1^ii^	0.99 (2)	2.968 (17)	3.8346 (16)	146 (1)
C16—H161···Cg2^ii^	0.99 (2)	2.685 (18)	3.5644 (16)	148 (1)
C16—H163···Cg3^ii^	1.00 (2)	2.643 (18)	3.5901 (15)	157 (1)

Symmetry codes: (i) −*x*+1, −*y*+1, −*z*+2; (ii) *x*, −*y*−1/2, *z*−3/2.
